# Efficacy and safety of damage control in experimental animal models of injury: protocol for a systematic review and meta-analysis

**DOI:** 10.1186/2046-4053-3-136

**Published:** 2014-11-22

**Authors:** Nela Cosic, Derek J Roberts, Henry T Stelfox

**Affiliations:** 1 Department of Critical Care Medicine, University of Calgary and the Foothills Medical Centre, 1403 29 Street Northwest, Calgary, Alberta T2N 2T9, Canada; 2 Department of Community Health Sciences, University of Calgary, 3280 Hospital Drive NW, Calgary, Alberta T2N 4Z6, Canada; 3 Department of Surgery, University of Calgary and the Foothills Medical Centre, 1403 29 Street Northwest, Calgary, Alberta T2N 2T9, Canada; 4 Department of Medicine, University of Calgary and the Foothills Medical Centre, 1403 29 Street Northwest, Calgary, Alberta T2N 2T9, Canada

**Keywords:** Animal experiments, Damage control surgery, Damage control interventions, Meta-analysis, Systematic review, Wounds and injuries

## Abstract

**Background:**

Although abbreviated surgery with planned reoperation (damage control surgery) is now widely used to manage major trauma patients, the procedure and its component interventions have not been evaluated in randomized controlled trials (RCTs). While some have suggested the need for such trials, they are unlikely to be conducted because of patient safety concerns. As animal studies may overcome several of the limitations of existing observational damage control studies, the primary objective of this study is to evaluate the efficacy and safety of damage control versus definitive surgery in experimental animal models of injury.

**Methods/design:**

We will search electronic databases (Medline, Embase, PubMed, Web of Science, Scopus, and the Cochrane Library), conference abstracts, personal files, and bibliographies of included articles. We will include RCTs and prospective cohort studies that utilized an animal model of injury and compared damage control surgery (or specific damage control interventions or adjuncts) to definitive surgery (or specific definitive surgical interventions). Two investigators will independently evaluate the internal and external/construct validity of individual studies. The primary outcome will be all-cause mortality. Secondary outcomes will include blood loss amounts; blood pressures and heart rates; urinary outputs; core body temperatures; arterial lactate, pH, and base deficit/excess values; prothrombin and partial thromboplastin times; international normalized ratios; and thromboelastography (TEG) results/activated clotting times. We will calculate summary relative risks (RRs) of mortality and mean differences (for continuous outcomes) using DerSimonian and Laird random effects models. Heterogeneity will be explored using subgroup meta-analysis and meta-regression. We will assess for publication bias using funnel plots and Begg’s and Egger’s tests. When evidence of publication bias exists, we will use the Duval and Tweedie trim and fill method to estimate the potential influence of this bias on pooled summary estimates.

**Discussion:**

This study will evaluate the efficacy and safety of damage control in experimental animal models of injury. Study results will be used to guide future clinical evaluations of damage control surgery, determine which animal study outcomes may potentially be generalizable to the clinical setting, and to provide guidelines to strengthen the conduct and relevance of future pre-clinical studies.

## Background

Hemorrhage is the leading preventable cause of death after injury worldwide [1-4]. Thus, identifying evidence-based methods of hemostasis is vitally important for improving trauma patient outcomes [1,4]. However, while randomized controlled trials (RCTs) have evaluated a range of medications and resuscitation strategies for bleeding trauma patients, few have reported improved patient outcomes [4,5]. As such, rapid and effective surgical or interventional hemostasis remains the most effective treatment [6].

The conventional surgical approach to managing traumatic hemorrhage is to definitively repair all injured organs in one operation [7]. However, as these repairs often require a significant amount of time, ongoing blood loss during surgery frequently leads to an inadequate blood supply to peripheral tissues resulting in anaerobic metabolism, lactic acidosis (low blood pH due to an accumulation of arterial lactate), and a decline in core body temperature [8,9]. Replacement of blood losses with crystalloid solutions devoid of clotting factors and platelets also often produces or worsens coagulopathy (i.e., abnormal blood clotting), which manifests as an absence of visible blood clots during surgery and/or abnormal clotting test results [7-9].

Trauma patients who are bleeding heavily therefore often develop a ‘lethal triad’ of hypothermia, acidosis, and coagulopathy, which has been linked with a high risk of mortality [7-10]. In order to shorten the duration of surgery and prevent death in those with coagulopathy, Stone et al. proposed abbreviated laparotomy with planned reoperation (later termed damage control laparotomy in 1993) [7,11,12]. Instead of attempting to repair all injuries during the index operation, this approach utilizes several abbreviated (or damage control) interventions [7,11,12]. These include application of compressive gauze packs to bleeding surfaces, tamponade of injured vascular structures with balloons (i.e., balloon catheter tamponade), and use of temporary intravascular shunts to bridge transected arteries and veins [6-9]. It also includes interventions that rapidly control spillage from wounds of the gastrointestinal organs, pancreas, and biliary tract [6,9].

As damage control laparotomy was associated with an increase in unexpected survivors during a time that the incidence of high energy and multicavitary penetrating and blunt trauma was rising in the United States and other countries, surgeons rapidly adopted this method over definitive laparotomy for management of severely injured patients [6,8]. The adoption of the procedure was further accelerated by the publication of several cohort studies, which reported that damage control surgery was associated with reduced mortality when appropriately applied [12-16]. As such, the procedure is now frequently utilized worldwide for management of major trauma patients [7].

Despite its potential advantages, damage control laparotomy is associated with several potentially severe complications, reduced quality of life, and significant costs to the health-care system [17-19]. Common complications include intra-abdominal abscesses and sepsis [17-19]. Approximately 8–29% of patients must also be managed with a ‘planned ventral hernia’ (in which the granulated abdominal viscera are covered only with a skin graft, resulting in a large hernia in the anterior part of the abdomen) [20-22]. Finally, there is an approximately 5% associated risk of development of enteroatmospheric fistulae, in which one or more intestinal segments communicate with the atmosphere, leading to significant patient distress, poor hygiene, ongoing intra-abdominal infection, and difficulty with nutrition and wound management [17-19].

As a result of the perceived lack of clinical equipoise among the surgical community, damage control has not been compared to definitive surgery in a RCT [7]. Although a recent Cochrane review suggested the need for such a trial, this is unlikely to be conducted due to patient safety concerns and the challenges associated with conducting a RCT of an emergent trauma surgical procedure [7,10]. Of the limited number of observational studies that have now directly compared the effectiveness of damage control and definitive surgery, the majority are small, potentially underpowered, and often based on retrospective reviews of databases maintained at single centers [7,10,23-26]. Further, results of these studies are frequently difficult to interpret due to varying inclusion/exclusion criteria, the lack of a complete description of which set of abbreviated trauma surgical procedures was studied, and the unavoidable presence of confounding by indication (whereby obvious or subtle differences exist in the characteristics of patients who are selected for damage control versus definitive surgery). Common baseline confounding variables in damage control effectiveness studies include the volume of resuscitation fluid provided and presenting blood pressures, core body temperatures, and acid/base and blood clotting measurements.

### Study rationale and objective

Animal models have the potential to avoid the limitations associated with retrospective reviews of human experiences through the use of stringent inclusion/exclusion criteria, standardized applications of interventions, randomization of animals to treatment groups, allocation concealment, and blinded assessments of outcome [27]. These types of studies may also be used to provide a biological foundation for surgical procedures [28,29]. Thus, the efficacy and safety of damage control may potentially be able to be informed by research conducted in animals [30-40].

In support of this, a large number of animal studies have evaluated the efficacy of damage control surgery and specific damage control interventions (e.g., rapid bowel ligation without reanastomosis [31,41], intraluminal balloon occlusion of the aorta [33,42], and temporary intravascular shunting of the superior mesenteric artery [30,39,40]) or adjuncts (e.g., commercial topical hemostatic agents for massively bleeding groin injuries [37,43]). See Table 1 for a complete list of damage control interventions/adjuncts that are currently used in clinical practice, including a sample of those that have been evaluated in animal models [30-32,39-41,43-53]. These studies may collectively provide insight into which interventions have the most promise for further clinical evaluation and use [27,29,54]. They may also reduce unwarranted animal use in future scientific studies and allow for the development of guidelines to strengthen the conduct and translational relevance of future pre-clinical trials [28,55].

**Table 1:** Examples of damage control interventions/adjuncts

**Interventions or adjuncts**	**Procedure**	**Examples**
Interventions	Thoracic	Therapeutic mediastinal and/or pleural space packing [6]; rapid lung-sparing surgery (pulmonary tractotomy, peripheral wedge resection, or pneumonorraphy) [7]; pulmonary hilum twisting [6]; and rapid stapled pneumonectomy
	Abdominal	Perihepatic or diffuse intra-abdominal packing [7]; peripancreatic drainage [7]; resection of a segment of bowel with delayed reanastomosis [7,8], rapid bowel ligation [31,41], or other techniques to control gastrointestinal spillage, including the use of skin staplers or allis clamps [6]; temporary abdominal closure techniques (including negative pressure peritoneal therapy or use of the Bogota bag [32]); and intraintestinal drainage
	Pelvis	Extraperitoneal pelvic packing [7]
	Vascular	Temporary intravascular shunting [30,39,40]; vascular ligation [30]; balloon catheter tamponade [9]; and arterial clamping [53]
Adjuncts	Interventions that assist in hemostasis	Topical hemostatic agents, including combat gauze or a fibrin patch [6,49,50] and modified rapid deployment hemostat bandage [44]; powders containing fibrin, collagen, and/or thrombin [44]; chitosan-based dressings [45]; absorbable mesh wraps/bandages [51,52]; zeolite powder-based dressings [43]; self-expanding, intracavitary polyurethane polymers [46]; and resuscitative balloon occlusion of the aorta (REBOA) [47].

Although the above animal studies likely have many advantages over currently published human studies, several potential biases must be critically examined before their results are attempted to be used to inform clinical research and practice [27,28,55,56]. These include those relating to construct validity, study design (e.g., sample size determination, randomization, and allocation concealment), and publication bias [55,57,58]. For the results of an animal study to potentially be translated to the clinical environment, the model must have construct validity (i.e., it must mimic the realities of human injury care) [55,57]. For example, animals with a major vascular injury should present with significant hemodynamic instability and abnormal physiology (e.g., hypothermia, acidosis, and/or coagulopathy). Further, interventions should be applied in a somewhat delayed, instead of immediate, fashion (in order to emulate the requisite time delays associated with transport of injured patients to a trauma center and preparation for surgical care) [59,60]. Authors should also base their analyses on sample size calculations, which provide assurance that animals have not been added to a study incrementally in response to interim analyses, and intention-to-treat principles [55,58,61]. Finally, as significant and positive results of animal studies are more likely to be published (and to be published in English-language journals), all of the available literature must be identified, regardless of language or origin of publication, and the potential influence of publication bias must be thoroughly assessed [55].

The objective of the proposed systematic review and meta-analysis is to compare the efficacy and safety of damage control surgery (or specific damage control interventions or adjuncts) to definitive surgery (or specific definitive surgical interventions) in experimental animal models of injury. We will also evaluate whether estimates of efficacy vary with the construct validity of the model and various study-level characteristics. In the presence of publication bias, we will use the Duval and Tweedie trim and fill method to determine the potential effect of adding a cohort of theoretically unpublished studies to summary estimates of efficacy.

## Methods/design

### Protocol

This protocol was created according to the Preferred Reporting Items in Systematic Reviews and Meta-analyses statement [62], the Meta-Analyses of Observational Studies in Epidemiology proposal [63], and the recommendations for reporting of systematic reviews and meta-analyses of animal experiments by Peters et al. [64] and Sena et al. [55]. As protocols for systematic reviews of animal studies are not currently eligible for inclusion in PROSPERO, this protocol has not been registered.

### Structured clinical question

#### Primary question

Is damage control surgery (or a specific damage control intervention) more efficacious in reducing mortality than definitive surgery (or a specific definitive surgical intervention) in experimental mammalian models of injury?

#### Secondary questions

1) Are particular damage control interventions (e.g., balloon catheter tamponade) more efficacious in reducing mortality than other damage control interventions in experimental mammalian models of injury?

2) Are particular damage control adjuncts (e.g., topical hemostatic agents) more efficacious in reducing mortality than use of no damage control adjunct or other damage control adjuncts in experimental mammalian models of injury?

### Search strategy

We will modify a sensitive search strategy previously developed for use in identifying studies of damage control surgery and damage control interventions [7]. With assistance from a medical librarian/information-scientist, we will search Ovid Medline and Embase, PubMed, Web of Science, Scopus, and the Cochrane Library without language, publication date, or other restrictions (Table 2 provides details of our planned bibliographic database search strategies). In order to increase the sensitivity of the search, we will also review the reference lists of all included and relevant review articles identified during the conduct of the search and use the PubMed ‘related articles’ and Google Scholar ‘cited by’ features. To identify studies that are about to be published, we will contact trauma surgery experts and relevant researchers in the field and conduct manual searches of abstracts for conferences held between 2009–2013, including meetings of the American Association for the Surgery of Trauma, American College of Surgeons, Eastern Association for the Surgery of Trauma, International Association for Trauma Surgery and Intensive Care, Trauma Association of Canada, and the Western Trauma Association [7].

**Table 2:** Details of electronic bibliographic database search strategies

**Ovid Medline and Medline In Process and Other Non-indexed Citations, PubMed, and the Cochrane Library**	**Ovid Embase**
1. damage control.ab,ti.	1. damage control.ab,ti.
2. (damage control adj3 (surg* or procedure* or approach* or laparotom* or celiotom*or thoracotom*)).ab,ti.	2. (damage control adj3 (surg* or procedure* or approach* or laparotom* or celiotom* or thoracotom*)).ab,ti.
3. (abbreviated adj3 (surg* or procedure* or laparotom* or celiotom* or thoracotom*)).ab,ti	3. (abbreviated adj3 (surg* or procedure* or laparotom* or celiotom* or thoracotom*)).ab,ti.
4. (staged adj3 (surg* or procedure* or approach* or laparotom* or celiotom* or thoracotom*)).ab,ti.	4. (staged adj3 (surg* or procedure* or approach* or laparotom* or celiotom* or thoracotom*)).ab,ti.
5. (bailout adj3 (surg* or laparotom* or celiotom* or thoracotom*)).ab,ti.	5. (bailout adj3 (surg* or laparotom* or celiotom* or thoracotom*)).ab,ti.
6. ((abdom* or thora* or chest* or liver* or hepatic) adj5 pack*).ab,ti.	6. ((abdom* or thora* or chest* or liver* or hepatic) adj5 pack*).ab,ti.
7. (balloon adj3 (tamponade or catheter tamponade)).ab,ti.	7. (balloon adj3 (tamponade or catheter tamponade)).ab,ti.
8. (temporary intravascular shunt* or intravascular shunt* or vascular shunt* or arterial shunt* or artery shunt*).ab,ti.	8. (temporary intravascular shunt* or intravascular shunt* or vascular shunt* or arterial shunt* or artery shunt*).ab,ti.
9. (topical hemostatic agent or combat gauze or fibrin patch or modified rapid deployment hemostat bandage)	9. (topical hemostatic agent or combat gauze or fibrin patch or modified rapid deployment hemostat bandage)
10. (fibrin or collagen or thrombin or zeolite or chitosan) adj3 (powder* or dressing*)	10. (fibrin or collagen or thrombin or zeolite or chitosan) adj3 (powder* or dressing*)
11. (intracavitary polyurethane polymer* or REBOA or resuscitative balloon occlusion)	11. (intracavitary polyurethane polymer* or REBOA or resuscitative balloon occlusion)
12. exp ‘Wounds and Injuries’/	12. exp injury/
13. exp Multiple Trauma/	13. exp blunt trauma/
14. exp Wounds, Penetrating/	14. exp multiple trauma/
15. exp Wounds, Gunshot/	15. exp stab wound/
16. exp Wounds, Stab/	16. exp missile wound/
17. exp Wounds, Nonpenetrating/	17. exp abdominal injury
18. exp Abdominal Injuries/	18. exp thorax injury/
19. exp Thoracic Injuries/	19. exp bleeding/
20. exp Hemorrhage/	20. (trauma* or multiple trauma* or polytrauma or penetrating trauma* or penetrating injur* or blunt trauma* or blunt injur* or injur* or wound* or stab* or gunshot*).ab,ti.
21. (trauma* or multiple trauma* or polytrauma or penetrating trauma* or penetrating injur* or blunt trauma* or blunt injur* or injur* or wound* or stab* or gunshot*).ab,ti.	21. 1 or 2 or 3 or 4 or 5 or 6 or 7 or 8 or 9 or 10 or 11
22. 1 or 2 or 3 or 4 or 5 or 6 or 7 or 8 or 9 or 10 or 11	22. 12 or 13 or 14 or 15 or 16 or 17 or 18 or 19 or 20
23. 12 or 13 or 14 or 15 or 16 or 17 or 18 or 19 or 20 or 21	23. 21 and 22
24. 22 and 23	

Table and search strategy modified from Roberts et al. [7]. ‘Where ab,ti. refers to abstract and title; exp to an exploded Medical Subject Heading term search; and adjX to an adjacency operator that will search for terms within X words of the key term of interest. The "*" indicates that a wildcard search will be performed: for example, in the case of surg*, surgery, surgical, and surgeries, among others, would all be searched for’ [7].

### Study selection

Two investigators (N.C., D.J.R.) will independently screen the titles and abstracts of all citations identified by the search and select those that may have involved animals and which mention use of damage control surgery, interventions, or adjuncts for full-text review. We will include studies: 1) that involved large (sheep, swine, and non-human primates, among others) or small (mice, rats, dogs, and cats, among others) animals in which injury was induced *in vivo* surgically or through an external blunt or penetrating force; 2) where the experimental intervention was damage control surgery, a damage control intervention, or a damage control adjunct; 3) where the comparator intervention was definitive surgery, a definitive surgical procedure, or an alternate type of damage control intervention or adjunct (or no damage control adjunct); and 4) where the study design was a RCT or prospective cohort study. Both RCTs and prospective cohort studies will be included as many potentially confounding factors are frequently controlled for in non-randomized animal studies. Injuries of interest will include those resulting in solid, hollow, and/or vascular organ damage in the trunk (neck, thorax, abdomen, or pelvis) as well as extremity vascular trauma [7,9]. For the purpose of this study, damage control surgery will be defined as ‘a multi-step operative intervention, which includes an abbreviated initial surgical procedure (or set of procedures) that aims to control obvious mechanical bleeding or contamination as compared to definitively repairing all injuries’ [7]. Definitive surgery will be defined as complete repair of the injury/injuries of interest at the index operation followed by formal closure of the explored cavity or region. In order to be as sensitive as possible, we will not predefine damage control interventions or damage control adjuncts. Instead, damage control interventions and adjuncts will be defined by study authors.

Agreement between investigators regarding identification of abstracts for full-text review and inclusion of articles in the systematic review will be quantified using kappa statistics [65]. Disagreements will be resolved by consensus between the two investigators.

#### Primary outcome

The primary outcome will be all-cause mortality measured at the longest follow-up time after administration of the intervention.

#### Secondary outcomes

Secondary outcomes will include volumes and/or rates of blood loss; laboratory surrogates of blood loss (complete blood counts, hemoglobin concentrations, and/or hematocrit values); and physiologic measurements, including blood pressures (mean arterial [MAP], systolic, and diastolic), heart rates, urinary outputs, core body temperatures, arterial lactate, base deficit/excess, and pH values, prothrombin and partial thromboplastin times, international normalized ratios, and thromboelastography (TEG) results/activated clotting times.

### Data extraction

Two investigators (N.C., D.J.R.) will independently extract data from included studies using a pre-designed data extraction spreadsheet. The design and reliability of the spreadsheet will be pilot tested on a random sample of an equal number of RCTs and prospective cohort studies until it is clear that the form captures all relevant information and consistency in data extraction is achieved (κ statistic ≥0.75) [65]. If a non-English language article appears potentially relevant (or if it is unclear if it is relevant), a single interpreter alone will review this article in full and complete data extraction if it satisfies inclusion/exclusion criteria. We will extract data from included studies on: 1) study characteristics; 2) characteristics of the included animals and the animal model, including the nature of the induced injury; 3) the treatment that was employed; and 4) mortality and secondary outcome measures (see Table 3 for a detailed description of the data to be collected) [66]. A RCT will be distinguished from a prospective cohort study using the criteria suggested by Oleckno [67].

**Table 3:** Relevant data extraction information

**Data extraction category**	**Specific information to extract**
Study characteristics	Author(s); year of publication, study design (randomized controlled trial or prospective cohort).
Characteristics of animal model	Species; strain; gender; total number of animals; number of animals in each experimental cohort; number of animals excluded; age (in total and per experimental cohort); weight (in total and per experimental cohort); and physiological characteristics reported by the authors at baseline before application of interventions (in total and per experimental cohort).
Details of injury induced in animals	Specifics of the injury that was induced; mechanism of producing the injury model (as described by authors); anatomical location of the injury or injuries (e.g., polytrauma model vs. single/more localized type of injury); and injury severity (e.g., specific grade of liver injury).
Details of treatment employed	Whether an entire damage control paradigm or an individual intervention or adjunct was tested; the specific treatment that was evaluated; and the definition of damage control surgery. Additional details collected are based on a modified version of the elements of experimental procedures outlined in the Animal Research: Reporting *In Vivo* Experiments (ARRIVE) guidelines: how, when, where, and why [66]: How? Specific method of employment of treatment tested and how long it was applied for. When? How long after injury was induced was the intervention(s)/adjunct(s) implemented? Where? Laboratory setting, surgical theater, or alternate setting as described by author(s). Why? To evaluate the purpose of testing the treatment.
Consequences of damage control surgery and/or definitive surgery to animals	Mortality; changes in post-injury physiological parameters, including estimated volumes and/or rates of blood loss, laboratory surrogates of blood loss (complete blood counts, hemoglobin concentrations, and/or hematocrits); and several physiologic measurements, including blood pressures (mean arterial (MAP), systolic, and diastolic), heart rates, urinary outputs, and core body temperatures, and arterial lactate, base deficit/excess, and pH values, prothrombin and partial thromboplastin times, international normalized ratios, and thromboelastography (TEG) results/activated clotting times.

### Risk of bias assessment

Two investigators (N.C., D.J.R.) will independently assess the internal and external/construct validity of included studies. Internal validity will be evaluated using a modified version of the ten-item Collaborative Approach to Meta-Analysis and Review of Animal Data from Experimental Studies (CAMARADES) checklist, which assesses the risk of study design-related biases in animal studies [57,62,68] (see below for a modified list of the CAMARADES checklist items to be assessed). A modified version of the Studies of Translation, Ethics, and Medicine (STREAM) checklist with additional factors incorporated from Lamontagne et al. and Krauth et al. will be utilized to evaluate construct validity [55,59,62,69] (see below for a modified list of the STREAM elements of interest to be assessed). The STREAM checklist is a tool designed to assess the internal, external, and construct validity of pre-clinical studies to determine their potential to be used to inform the design of clinical trials [55,69]. To further evaluate external validity, we will assess whether the same conclusions were found in different studies that induced the same injury model and employed the same treatment in different types of animals (species or strain) [55,69].

### Modified CAMARADES checklist items for assessing internal validity of individual studies [56,61]

▪ Publication in a peer-reviewed journal instead of only in abstract form

▪ Randomization of treatment

▪ Presence of allocation concealment

▪ Blinded assessment of outcome

▪ Conduct of a pre-experimentation sample size calculation

▪ Evidence of animal welfare compliance

▪ Statement regarding possible conflict of interest

▪ Completeness of follow-up

The checklist items ‘statement of control of temperature’ and ‘use of animals with hypertension or diabetes’ were removed from the above list as they were developed for assessing animal studies of stroke interventions [56,67]. The item ‘avoidance of anesthetic with marked intrinsic properties’ was also removed as it will be addressed in the assessment of construct validity [54,56,69]. The item ‘completeness of follow-up’ was added to the list as per guidelines proposed by Lamontagne et al. [61].

### Modified STREAM checklist items for assessing construct validity of individual studies [56,58,61,69]

▪ The extent that the injury induced in the animal model accurately represents the realities of the injury in humans

▪ The extent that the intrinsic characteristics of the animals chosen represent the civilian population that is most likely to receive damage control

▪ Relevance of treatment tested to the clinical setting

▪ Evaluation of whether treatments were employed accurately/as they would in the clinical setting

▪ Explicit statement that an intention-to-treat analysis was used

▪ Explicit statement of inclusion reason(s) for animals utilized in study

▪ Explicit statement of exclusion reason(s) for animals removed from analysis of results

▪ Explicit statement in study addressing potential confounding factors/sources of bias

▪ Evidence of delay in employment of treatment to emulate realities of emergent surgical care

▪ Evidence of a pre-specified stopping rule

▪ Reporting of a potentially patient-important outcome such as mortality

The clinical relevance of the study design will be assessed independently by two fellowship-trained trauma surgeons. For the item related to providing evidence of delay in employment of treatment, if the intervention was applied in the ambulance, the delay should be approximately 20 min to account for a 5-min response time and about a 15-min on scene time [60]; if the intervention was applied in the Emergency Department, the delay should be approximately 30 min [59,60]; and if the intervention was applied in the operating room, the delay should be approximately 45 min [59].

### Data synthesis

Both a narrative synthesis and, where possible, a meta-analysis of the results of the individual studies will be presented. Studies will first be grouped according to the types of interventions and controls studied. After studies have been combined into common groups, their characteristics will be presented in summary tables. These groupings will be utilized to determine where categories of studies exist that have similar enough designs to allow for a meta-analysis of their reported outcomes.

### Statistical analyses

To address the primary research question, we will calculate individual study estimates of the relative risk (RR) comparing mortality between animals that received damage control (or a specific damage control intervention) versus definitive surgery (or a definitive surgical intervention). Similarly, for the secondary research questions, we will calculate RRs comparing mortality between animals that received different damage control interventions or adjuncts or a damage control adjunct versus no adjunct. As we expect that there will be ‘clinical’ heterogeneity in the design and/or conduct of the included studies, individual study RR estimates will be combined using DerSimonian and Laird random effects models [70]. However, in the event that we find little clinical heterogeneity between studies, we will combine individual study RR estimates using fixed effect models with Mantel-Haenszel weighting [71]. Where possible, for both the primary and secondary research questions, we will also use random effects or fixed effect models and the reported within-group mean values to calculate summary mean differences for the above secondary outcome variables.

We will examine for evidence of between-study heterogeneity by calculating *I*^2^ inconsistency values and conducting Cochran’s Q hypothesis tests of homogeneity (*p* value <0.1 considered significant given the low power of these tests). Heterogeneity will be defined according to the value of the *I*^2^ statistic as low (*I*^2^ > 25%), moderate (*I*^2^ > 50%), and high (*I*^2^ > 75%) [72]. In the presence of heterogeneity, we will conduct stratified meta-analyses and meta-regression to determine whether our summary estimates vary across different study-level covariates. Covariates of interest will include characteristics of study internal validity, including whether randomization of animals to treatment was performed, allocation was concealed, or a sample size calculation was reported to have been done [57]. Other covariates will include characteristics of external/construct validity, including whether an intention-to-treat analysis was used, whether potential confounding factors were well balanced between the groups at baseline or controlled for, or whether there was a delay in employment of the treatment of interest by at least 30 min for an intervention designed to be applied in the Emergency Department versus 45 min for one designed to be used in the operating room [55,59,62,69]. Important confounding variables that should have been considered by study authors include baseline blood pressures, core body temperatures, arterial pH values, and laboratory clotting test results.

We will assess for the presence of small study effects potentially due to publication bias by plotting funnel plots of the RR of mortality. Evidence for the existence of funnel plot asymmetry will be assessed using Begg’s funnel plot asymmetry test and confirmed using Egger’s regression test [73,74]. When evidence of publication bias exists, we will use the Duval and Tweedie ‘trim and fill’ method to estimate the potential influence of this bias on our pooled summary estimates [75,76]. In this method, the small outlying study results are first ‘trimmed’ (removed until the funnel plot appears symmetrical) and then the remaining symmetric study results are used to re-estimate the ‘true’ center of the plot [75-77]. Subsequently, the plot is ‘filled’ (the missing outlying study results and their theoretical ‘counterparts’ are replaced around the re-estimated center) and an adjusted pooled estimate is calculated [75-77].

## Discussion

Animal experiments are frequently used to evaluate interventions before they are used clinically. Although animal studies provide evidence of the performance of interventions under relatively ideal conditions, some are limited by study design-related biases and/or a lack of model construct validity. Thus, systematic reviews of these types of studies may be used to outline which interventions tested in animal models do not yet have enough supporting evidence to warrant evaluation in clinical trials [29,55-57,78]. Unfortunately, it has been reported that this may infrequently occur in practice. For example, in a systematic review of 1,026 experimental treatments for acute ischemic stroke, the drugs that were ultimately evaluated clinically were no more effective in animal models than those not evaluated clinically [78].

Systematic reviews of animal intervention studies are therefore important to guide the transition of scientific findings from the bench to the bedside [78]. These reviews may be used to determine that interventions with consistent positive outcomes across several experimental studies may safely be generalized to the design and conduct of clinical trials [29,30,55,79]. For example, a systematic review of the effect of hypothermia in acute ischemic stroke demonstrated that infarct size was consistently reduced across all animal models when core body temperature was cooled to approximately 35°C [79]. There have since been multiple clinical trials (with many still in progress) evaluating the effect of cooling in acute ischemic stroke patients, including the Nordic Cooling Stroke Study (NOCSS) and the Cooling in Acute Stroke-II (COAST-II) study [80,81].

Systematic reviews of animal studies have also influenced the conduct of pre-clinical trials. Several systematic reviews have suggested how the design, construct validity, methodological rigor, and reporting of animal studies may be improved such that their relevance to human healthcare is enhanced [56,57,82]. In addition, the compilation of world literature regarding pre-clinical animal models outlines which studies have already been conducted in animals, thereby potentially preventing wasteful allocation of future resources to studies that will be ineffective or repetitive (and protecting animals from potentially unwarranted future experimentation) [30].

Through this systematic review and meta-analysis, we seek to establish whether damage control is more efficacious than definitive surgery and if so, which damage control interventions are more efficacious than others. To accomplish this, we will assemble all original scientific research examining the efficacy and safety of damage control in experimental animal models of injury. After considering animal model construct validity, study design-related biases, and whether publication bias may have existed, we will determine which damage control interventions or adjuncts may potentially provide valid and generalizable evidence for further clinical evaluation and suggest how to improve future pre-clinical trials. Thus, our results may be used to both inform clinical research and practice and more appropriately direct future research conducted in animals.

## Abbreviations

RCT: randomized controlled trial; CAMARADES: Collaborative Approach to Meta-Analysis and Review of Animal Data from Experimental Studies; STREAM: Studies of Translation, Ethics and Medicine; TEG: thromboelastography; MAP: mean arterial blood pressure; REBOA: resuscitative balloon occlusion of the aorta; RR: relative risk; ICU: intensive care unit; AIHS: Alberta Innovates-Health Solutions; ARRIVE: Animal Research: Reporting *In Vivo* Experiments.

## Competing interests

The authors declare that they have no competing interests.

## Authors’ contributions

NC drafted the manuscript. NC and DJR contributed to the drafting of the manuscript, formulation of the study selection criteria and risk of bias assessments, and the compiling of tables and the data extraction spreadsheet. DJR and HTS were responsible for formulating the search and for the development of the study design, research question, and statistical analysis/synthesis of data plan. All authors contributed to manuscript revisions and reviews of numerous drafts and agreed upon a final version.

## Authors’ information

NC is an undergraduate Bachelor of Science program student at the University of Calgary. DJR is a surgery and Clinician Investigator Program resident and Surgeon Scientist Program member who is currently pursuing a Doctor of Philosophy degree in Epidemiology and Knowledge Translation with a thesis on trauma damage control surgery at the University of Calgary. HTS is an academic intensive care physician who has an interest in health services research related to critically ill patients.
